# Cohort selection and the estimation of racial disparity in mortality of extremely preterm neonates

**DOI:** 10.1038/s41390-023-02766-0

**Published:** 2023-08-14

**Authors:** Jeffrey B. Gould, Mihoko V. Bennett, Jochen Profit, Henry C. Lee

**Affiliations:** 1 Department of Pediatrics (Neonatology), Stanford University, Stanford, CA USA; 2 California Perinatal Quality Care Collaborative, Stanford, CA USA; 3 University of California San Diego, La Jolla, CA USA

## Abstract

**Background:**

Racial disparities in preterm neonatal mortality are long-standing. We aimed to assess how cohort selection influences mortality rates and racial disparity estimates.

**Methods:**

With 2014–2018 California data, we compared neonatal mortality rates among Black and non-Hispanic White very low birth weight (VLBW, <1500 g) or very preterm infants (22–29 weeks gestational age). Relative risks were estimated by different cohort selection criteria. Blinder-Oaxaca decomposition quantified factors contributing to mortality differential.

**Results:**

Depending upon standard selection criteria, mortality ranged from 6.2% (VLBW infants excluding first 12-h deaths) to 16.0% (22–29 weeks’ gestation including all deaths). Black observed neonatal mortality was higher than White infants only for delivery room deaths in VLBW infants (5.6 vs 4.2%). With risk adjustment accounting for higher rate of low gestational age, low Apgar score and other factors, White infant mortality increased from 15.9 to 16.6%, while Black infant mortality decreased from 16.7 to 13.7% in the 22–29 weeks cohort. Across varying cohort selection, risk adjusted survival advantage among Black infants ranged from 0.70 (CL 0.61–0.80) to 0.84 (CL 0.76–0.93).

**Conclusions:**

Standard cohort selection can give markedly different mortality estimates. It is necessary to reduce prematurity rates and perinatal morbidity to improve outcomes for Black infants.

**Impact:**

- In this population-based observational cohort study that encompassed very low birth weight infant hospitalizations in California, varying standard methods of cohort selection resulted in neonatal mortality ranges from 6.2 to 16.0%.
- Across all cohorts, the only significant observed Black-White disparity was for delivery room deaths in Very Low Birth Weight births (5.6 vs 4.2%).
- Across all cohorts, we found a 16–30% survival advantage for Black infants.
- Cohort selection can result in an almost three-fold difference in estimated mortality but did not have a meaningful impact on observed or adjusted differences in neonatal mortality outcomes by race and ethnicity.

## Introduction

Decreasing the extent of racial and ethnic disparity is an important goal for perinatal medicine.^1–4^ In estimating Black to White differences in neonatal mortality among extremely prematurely born infants, several possibilities in cohort specification arise. Should the lower gestational age be set at 24 or 22 weeks, or should all very low birth weight (VLBW, < 1500 grams) infants be included regardless of gestational age? Should all deaths prior to discharge from the NICU be included? Or should exclusions be applied to delivery room deaths, all deaths before 12 h, and/or infants with congenital anomalies? The goal of using these various criteria is to create more homogeneous comparison groups.^5^ For example, while the approach to initiating care for all infants at 24 or more weeks of gestation is widely accepted, the initiation of the full extent of intensive care before 24 weeks varies across institutions. Excluding infants less than 24 weeks has been used to avoid this source of variability. Similarly, delivery room deaths and deaths in the first 12 h have been excluded to avoid potential institutional differences in perinatal care affecting an infant’s status at birth, approaches to resuscitation, and decisions for comfort care.^6^ Congenital anomalies are often excluded to avoid the complexity of their risk adjustment, in the hopes of creating a more level playing field for comparison. In addition, because the VLBW designation has had long and familiar use, some investigators prefer selecting their study cohorts based on birth weight rather than gestational age.

Although these various selection criteria have been widely used, we know of no systematic evaluation of their impact on estimating the rate of neonatal mortality. Furthermore, an important possibility is that these selection criteria could also influence the extent of the Black–White disparity. For example, with respect to the choice of gestational age, the extent of Black–White disparity has been shown to increase with gestational age and birth weight.^7–9^ Anderson et al. report that at 22–25 weeks gestational age, Black infants were less likely to die than White infants (odds ratio 0.76) while they were more likely to die at 32–34 weeks (odds ratio 1.64) and 35–36 weeks (odds ratio 1.57). Lee et al. commenting on the estimates published by the National Center for Health Statistics, state that standardization (such as 22 weeks) with a minimal gestational age could potentially mask the disproportionate contributions of previable births to infant mortality rates among the Black population which has the largest proportion of previable births.^10^ These findings suggest that estimates of the extent of Black–White disparity could differ depending on the choice of both the lower cut point and the range of gestational age.

The timing of death has also been shown to influence the estimate of racial disparity, with Black neonatal mortality being slightly higher in the first week of life (relative risk 2.3) than the remainder of the first month (relative risk 2.1).^8^ Although we have been unable to identify reports of racial differences in delivery room deaths in the United States, a study from Japan found that socioeconomic factors substantially influenced whether births of 22–24 weeks gestational age survive delivery and the first hour after birth.^11^ If this holds true in the U.S.A., excluding delivery room deaths could bias disparity estimates by ignoring a potential excess of delivery room deaths amongst Black infants. Furthermore, excluding an excess of deaths in the delivery room could result in a healthier post-delivery cohort with a lowered observed mortality due to delivery room “culling.”

Basing a cohort on weight (less than 1500 g) rather than gestational age could also affect estimating disparity. In a comparison between outcomes in a cohort based on gestational age <32 weeks vs a cohort with birth weight <1500 g, neonatal mortality rates were slightly higher; furthermore, small for gestational age rates were markedly higher (9.3 vs 20.4%) in the weight-based cohort.^12^ Given that small for gestational age is associated with increased mortality and births amongst Black families have double the number of small for gestational age births when compared to White families, the extent of disparity estimated from a weight based cohort could differ from that of a cohort based on gestational age.^13^

In addition to gestational age vs weight, and timing of death, the inclusion or exclusion of neonates with severe congenital anomalies could influence the estimation of disparity in that mortality due to severe congenital anomalies is higher in Black than in White neonates (107.2 vs 133.1 per 100,000).^8^

The purpose of this study was to systematically assess the potential impact of cohort selection on both the overall estimation of very preterm mortality and the estimation of observed and risk adjusted differences in very preterm mortality for Black compared to White infants. Specifically, we compared overall mortality for all very preterm births as well as the Black–White disparity estimated from four cohorts: 24–29 weeks gestational age without congenital anomalies, 22–29 weeks without congenital anomalies, 400–1500 g without congenital anomalies, and 400–1500 g with congenital anomalies. For each cohort, we also assessed the impact of excluding delivery room deaths and deaths in the first 12 h. Based on the observations described above, we hypothesize that in any selected cohort, both the rate of overall mortality and the extent of racial disparity may differ between the infants that are included and those that are excluded from the cohort. Such differences may bias estimates of both overall mortality as well as the extent of racial disparity.

## Methods

The study cohort included VLBW and very preterm infants (less than 32 weeks) admitted into California NICUs between 2014 and 2018 among 140 hospitals in the California Perinatal Quality Care Collaborative (CPQCC). CPQCC has standardized data collection across member hospitals with variable definitions aligned with the Vermont Oxford Network.

The significance of differences in the race specific rates derived from the cohort specifications were assessed by Chi Square. We derived overall mortality and rates stratified by self-identified maternal race and ethnicity, and estimated the observed relative risk (RR) with its 95% asymptotic Wald confidence limits (CL). The significance test for mortality rates between non-Hispanic White (White) and Black infants accounted for multiple testing correction adjustment based on permutation tests.^14,15^ Patient risk profiles were compared using the chi-squared test among White and Black patients.

Multivariable analysis was performed using a log-binomial regression model controlling for risk factors associated with neonatal mortality. Our models included biologic variables (gestational age, multiple gestation, sex, small for gestational age), as well as potentially modifiable risk factors; a demographic/socioeconomic factor (maternal age),^4^ maternal health conditions (hypertension, diabetes, chorioamnionitis), maternal care factors (prenatal care, cesarean delivery, antenatal steroids, location of birth), and the infant’s condition at birth (Apgar score at 5 min), a factor influenced by maternal health and the quality of perinatal care, based on all race and ethnicity groups in the database. Individual hospital factors were not used in our modeling. The risk-adjusted mortality rate for each race was estimated by race-specific observed mortality rate divided by its expected rate to obtain the observed to expected ratio. Then the observed to expected ratio was multiplied by the overall mortality rate for the modeling population. The risk adjusted relative risk (aRR) for Black infants compared with White infants was estimated from a log-binomial model including race and the above risk factors.

Blinder–Oaxaca decomposition was used to further investigate, identify, and quantify factors that contributed to the mortality differential for Black and White infants born at 22–29 weeks of gestation without significant congenital anomalies.^16^ This technique assesses the differences in mortality rates by decomposing the gap into differences among the rates of each of the identified risk factors and the strength of association of each risk factor with the outcome. For example, the technique has been used to deconstruct racial disparity in preterm birth,^17^ birth outcomes,^18^ postpartum readmission rates,^19^ and poverty and affluence.^20^

This study was reviewed and approved by the Stanford University institutional review board. Analyses were performed using SAS 9.4 (SAS Institute) and Stata 15.1 (StataCorp).

## Results

### Impact of cohort selection on rates of observed neonatal mortality

The impact of how decisions of cohort inclusion based on gestational age, or based on birth weight regardless of gestational age, may affect the estimation of observed neonatal mortality in very preterm infants was examined. Among California births in 2014–2018, cohorts were established as follows: 24–29 weeks gestational age, 22–29 weeks gestational age, birth weight 401–1500 grams, and with or without congenital anomalies (Table 1). Beginning with the 14,205 infants born at 24–29 weeks of gestation without congenital anomalies, we identified 147 delivery room deaths (1%), 171 NICU deaths in the first 12 h (1.2%) and 1025 deaths from 13 h onward (7.2%) for a total of 1343 (9.5%) in-hospital deaths. If the 147 delivery room deaths and the 171 deaths in the first 12 h are excluded, the neonatal mortality estimate is reduced from 9.5% to 7.2% (Table 1).

**Table 1:** All race/ethnicity neonatal mortality by cohort selection criteria.

	Infant counts	Delivery room deaths		Died 12 h^a^		Died 13 h–discharge^b^		All deaths	
		*n*	%	*n*	%	*n*	%	*n*	%
24–29 GA weeks	14,205	147	1.0	171	1.2	1025	7.2	1343	9.5
Add 22–< 24 weeks	1710	745	43.6	83	4.9	382	22.3	1210	70.8
									
22–29 GA weeks	15,915	892	5.6	254	1.6	1407	8.8	2553	16.0
Add 401–1500 g, not 22–29 weeks	7941	232	2.9	13	0.2	68	0.9	313	3.9
									
401–1500 g or 22–29 GA weeks	23,856	1124	4.7	267	1.1	1475	6.2	2866	12.0
Add congenital anomaly	1715	243	14.2	78	4.5	300	17.5	621	36.2
									
401–1500 g or 22–29 GA weeks incl. congenital anomaly	25,571	1367	5.3	345	1.3	1775	6.9	3487	13.6

^a^Died in the first 12 h after surviving the delivery room.

^b^Died prior to discharge after surviving the first 12 h after birth.

Next, 1710 infants with gestational age (GA) of 22 to less than 24 weeks were added, increasing the size of the cohort to 15,915 infants born at 22–29 weeks. The overall mortality of these 1710 additional infants was high (70.8%) in large part due to the 745 delivery room deaths (43.6% mortality) and the 382 deaths (22.3% mortality) from 13 h until discharge. The addition of these high-risk infants increased the overall cohort mortality from 9.5% using the 24–29 weeks definition to 16.0% using the 22–29 weeks definition. The most dramatic increase in mortality was seen in delivery room deaths which increased from 147 (1.0%) to 892 (5.6%) (Table 1).

To create the VLBW cohort, 7941 very low birth weight infants (401–1500 g) were added whose gestational age did not fall in the previously included 22–29 weeks range. The size of this VLBW cohort increased to 23,856. These additional infants were mostly born at gestational age greater than 29 weeks. The added 7941 infants had an overall mortality of only 3.9% and constituted a third of the VLBW cohort. Their addition led to a decrease in the expanded cohort’s mortality from 16.0 to 12.0%. The rate of delivery room deaths also fell from 5.6% to 4.7% (Table 1).

When the 1715 infants with significant congenital anomalies were added to the VLBW cohort, infants with high rate of neonatal death (36.2%), the overall observed mortality increased from 12.0 to 13.6% and delivery room deaths increased from 4.7 to 5.3% (Table 1).

If we consider all >22 week VLBW births (*n* = 25,571) and deaths (1775) as a maximum count of very preterm births and deaths, the 24–29 week cohort accounts for 56% of the births and 38.5% of the deaths, the 22–29 week cohort 62% of the births and 73.2% of the deaths, and the VLBW cohort without congenital anomalies, 93% of the births and 82.2% of the deaths.

Commonly employed cohort selection criteria and the decision to include or exclude delivery room and deaths in their first 12 h can result in very preterm mortality rates as low as 6.2% and as high as 16.0% (Table 1). In all four cohorts described above, mortality that excluded delivery room and deaths in the first 12 h of life was markedly lower than mortality that included all deaths, 7.2 vs 9.5% (24–29 weeks), 8.8 vs 16.0% (22–29 weeks), 6.2 vs 12.0% (VLBW), and 6.9 vs 13.6% (VLBW including congenital anomalies). All of the rate comparisons shown in Table 1 are statistically significant (*p* < 0.001).

### Potential impact of cohort selection on estimates of observed differences in neonatal mortality in Black and White preterm infants

When the comparisons of the cohorts presented above were limited to include only Black and White infants, the results were similar, with mortality rates ranging from 6.2% (deaths from 12 h to discharge in the VLBW cohort) to 16.1% (all deaths in 22–29 weeks cohort) (Appendix Table 6). To assess the impact of cohort selection on estimating potential racial differences in observed neonatal mortality, the RR for each of the four cohorts were estimated based on all deaths, the exclusion of delivery room deaths, and the exclusion of deaths in the first 12 h (Table 2). When corrected for multiple comparisons, the only statistically significant Black–White difference was seen in the delivery room deaths (5.6 vs 4.2%) of the VLBW cohort. All deaths in VLBW cohort (11.3 vs 12.9%, RR = 1.14, CL = 1.02–1.28) and delivery room deaths in 22–29 weeks (5.4 vs 6.8%) indicated increased Black infant mortality, but their differences were no longer statistically significant after multiple testing adjustment.

**Table 2:** The effect of cohort selection on the rates and comparison of Black and White non-Hispanic neonatal mortality.

Infant count	Race	Delivery room death		Died before 12 h		Died at discharge		All deaths		
		*n*	%	*n*	%	*n*	%	*n*	%	Observed relative risk
*Gestational age 24–29 weeks (24 0/7–29 6/7)*										
All										
3491	W	33	0.9%^a^	37	1.1%^a^	262	7.5%^a^	332	9.5%^a^	Ref
1862	B	22	1.2%	20	1.1%	129	6.9%	171	9.2%	0.97 (0.81, 1.15)
Excluding delivery room deaths										
3458	W	–	–	37	1.1%	262	7.6%	299	8.6%	Ref
1840	B	–	–	20	1.1%	129	7.0%	149	8.1%	0.94 (0.77, 1.13)
Excluding delivery room death and death before 12 h										
3421	W	–	–	–	–	262	7.7%	262	7.7%	Ref
1820	B	–	–	–	–	129	7.1%	129	7.1%	0.93 (0.75, 1.13)
*Gestational age 22–29 weeks (22 0/7–29 6/7)*										
All										
3853	W	208	5.4%^a^	54	1.4%^a^	350	9.1%^a^	612	15.9%^a^	Ref
2145	B	146	6.8%^b^	29	1.4%	181	8.4%	356	16.6%	1.04 (0.92, 1.17)
Excluding delivery room deaths										
3645	W	–	–	54	1.5%	350	9.6%	404	11.1%	Ref
1999	B	–	–	29	1.5%	181	9.1%	210	10.5%	0.95 (0.81, 1.11)
Excluding delivery room death and death before 12 h										
3591	W	–	–	–	–	350	9.7%	350	9.7%	Ref
1970	B	–	–	–	–	181	9.2%	181	9.2%	0.94 (0.79, 1.12)
*401–1500* *gm or 22–29 weeks gestational age (“VLBW”, no congenital anomaly)*										
All										
5980	W	251	4.2^a^	57	1.0^a^	365	6.1^a^	673	11.3^a^	Ref
3081	B	174	5.6^c^	30	1.0	193	6.3	397	12.9	1.14^b^ (1.02, 1.28)
Excluding delivery room deaths										
5729	W	–	–	57	1.0%	365	6.4%	422	7.4%	Ref
2907	B	–	–	30	1.0%	193	6.6%	223	7.7%	1.04 (0.89, 1.22)
Excluding delivery room death and death before 12 h										
5672	W	–	–	–	–	365	6.4%	365	6.4%	Ref
2877	B	–	–	–	–	193	6.7%	193	6.7%	1.04 (0.88, 1.23)
*401–1500* *g or 22–29 weeks gestational age (“VLBW”) including congenital anomaly*										
All										
6454	W	318	4.9^a^	73	1.1^a^	449	7.0^a^	840	13.0^a^	Ref
3246	B	191	5.9^b^	37	1.1	226	7.0	454	14.0	1.07 (0.96, 1.19)
Excluding delivery room deaths										
6136	W	–	–	73	1.2%	449	7.3%	522	8.5%	Ref
3055	B	–	–	37	1.2%	226	7.4%	263	8.6%	1.01 (0.88, 1.16)
Excluding delivery room death and death before 12 h										
6063	W	–	–	–	–	449	7.4%	449	7.4%	Ref
3018	B	–	–	–	–	226	7.5%	226	7.5%	1.01 (0.86, 1.18)

Note: With the exception of deliver room deaths in the 401–1500 g or 22–29 GA cohort without congenital anomaly, after correcting for multiple testing, all of the racial comparisons shown in the table did not reach statistical significance.

^a^Reference.

^b^Raw *p* value is statistically significant, but not statistically significant with multiple testing adjustment.

^c^Statistically significant after multiple testing adjustment.

When cohort selection was based solely on gestational age (22–29 or 24–29 weeks), no significant racial disparities were observed when RR estimates were based on the entire cohort, based on when delivery room deaths were excluded, and based on when deaths in the first 12 h were excluded. These six observed RRs ranged from 0.93 (CL 0.75–1.13) to 1.04 (CL 0.92–1.17) (Table 2).

### Potential impact of cohort selection on estimates of risk adjusted differences in neonatal mortality in Black and White preterm infants

Risk adjusted rates were estimated based on a log binomial model that included all race groups. For each of the four cohorts, racial differences based on including all deaths and excluding delivery room deaths and deaths in the first 12 h of life were estimated. Observed and risk adjusted rates and their RRs are compared in Table 3. In contrast to observed rates where the only racial difference was a Black disadvantage seen in Delivery room deaths in the VLBW cohort without congenital abnormalities, for all the cohort comparisons made on risk adjusted rates, Black infants had significantly lower rates of neonatal mortality than White infants. Furthermore, in all four cohorts, when delivery room and early deaths are excluded, the risk adjusted Black infant mortality advantage was slightly increased. For example, in the 22–29 weeks cohort without congenital anomalies, the risk adjusted aRR estimate went from a 16% advantage (aRR = 0.84, CL 0.76–0.93) including all deaths to a 27% advantage (aRR = 0.73, CL 0.63–0.85) when delivery room deaths and deaths in the first 12 h were excluded. Although this increase did not reach statistical significance, it raises the possibility that delivery room survival and survival in the first 12 h are being impacted by unknown factors such as the wishes of the family, the decisions/protocols of the physicians, or the quality of care, whose identification could provide opportunities for quality improvement.

**Table 3:** The effect of cohort selection on the risk adjusted comparison of Black and White non-Hispanic neonatal mortality.

		Number	Observed mortality	Observed relative risk (95% confidence interval)	Adjusted mortality	Adjusted relative risk (95% confidence interval)
24–29 weeks						
All infants	White	3491	9.5%	Ref	10.2%	Ref
	Black	1862	9.2%	0.97 (0.81, 1.15)	8.3%	0.85 (0.72, 0.99)
Excluding delivery room death and death before 12 h	White	3421	7.7%	Ref	8.2%	Ref
	Black	1820	7.1%	0.93 (0.75, 1.13)	6.3%	0.79 (0.65, 0.96)
22–29 weeks						
All infants	White	3853	15.9%	Ref	16.7%	Ref
	Black	2145	16.6%	1.04 (0.92, 1.17)	13.7%	0.84 (0.76, 0.93)
Excluding delivery room death and death before 12 h	White	3591	9.7%	Ref	10.7%	Ref
	Black	1970	9.2%	0.94 (0.79, 1.12)	8.0%	0.73 (0.63, 0.85)
401–1500 g or 22–29 weeks						
All infants	White	5980	11.3%	Ref	12.3%	Ref
	Black	3081	12.9%	1.14 (1.02, 1.28)	10.4%	0.83 (0.74, 0.93)
Excluding delivery room death and death before 12 h	White	5672	6.4%	Ref	7.3%	Ref
	Black	2877	6.7%	1.04 (0.88, 1.23)	5.7%	0.7 (0.59, 0.81)
401–1500 g or 22–29 weeks						
Including congenital anomaly	White	6454	13.0%	Ref	13.8%	Ref
	Black	3246	14.0%	1.07 (0.96, 1.19)	11.9%	0.83 (0.75, 0.91)
Excluding delivery room death and death before 12 h	White	6063	7.4%	Ref	8.3%	Ref
	Black	3018	7.5%	1.01 (0.86, 1.18)	6.6%	0.7 (0.61, 0.80)

Note. Risk adjustment model is based on log binomial model including all race groups.

*Ref* reference.

### Accounting for the difference in risk adjusted vs observed rates

Table 4 shows comparisons of the frequencies of known risk factors used in risk adjusted comparisons of Black and White neonatal mortality in the 22–29 weeks cohort without congenital anomalies. Black infants have higher levels of risk with respect to the percentage of low gestational age, small for gestational age, low rates of antenatal steroids, low 5-min Apgar scores, teenage birth, and maternal chorioamnionitis. However, Black infants also had lower levels of risk with respect to the percent of male births, multiple births, cesarean, and outborn deliveries. The Blinder–Oaxaca decomposition procedure was used to assess the impact of these differing levels of risk. Using this approach, the difference in the rates of two groups can be partitioned into differences in the frequency of their common risk factors, and differences in the strength of the relationship between each of these risk factors and the outcome.^16^ We performed this analysis in the 22–29 week cohort which excluded 72 (1.2%) infants who had a missing risk variable (Appendix Table 7). The observed mortality was 15.9% in White vs 16.6% in Black infants for an absolute gap of 0.7% in favor of White infants. Following risk adjustment based on the variables shown in Table 4, White neonatal mortality was increased to 16.7% and Black neonatal mortality was decreased to 13.7%, an absolute gap of 3.0% in favor of Black infants. Table 5 summarizes the contributions of differences in the percent of risk factors and differences in the strength of each risk factor to this risk adjusted gap. With respect to “endowments,” the most important risk differences were the increased levels of low gestational age and low 5-min Apgar scores experienced by Black infants. These two risk factors accounted for 89.8% of the risk adjusted gap. There were also some risk levels that were lower in Black infants such as fewer multiple births and cesarean births. Unlike the differences seen in the contributions of the “endowments”, most risk factors had similar strengths of association with neonatal mortality for Black and White infants. The one exception was the impact of being outborn, which was lower in White births and lowered the gap by 0.1%. (Table 5 and Appendix Table 8). In summary, risk adjustment revealed that Black infants have an apparent survival advantage that was masked by their higher levels of risk factors associated with mortality.

**Table 4:** Risk profile of Black and White infants with gestational age 22–29 weeks without congenital anomalies.

Risk factor		Non-Hispanic White			Black	
		*n*/*N*	%	*n*/*N*	%	*P* value^b^
Birth weight^a^ (g)	400 or less	40/3853	1.0%	36/2145	1.7%	<0.0001
	401–750	979/3853	25.4%	731/2145	34.1%	–
	751–1000	1016/3853	26.4%	627/2145	29.2%	–
	1001–1250	989/3853	25.7%	479/2145	22.3%	–
	1251–1500	634/3853	16.5%	223/2145	10.4%	–
	1501+	195/3853	5.1%	49/2145	2.3%	–
Gestational age week	22–23	362/3853	9.4%	283/2145	13.2%	<0.0001
	24–25	724/3853	18.8%	494/2145	23.0%	–
	26–27	1073/3853	27.8%	601/2145	28.0%	–
	28–29	1694/3853	44.0%	767/2145	35.8%	–
Small for gestational age		393/3853	10.2%	283/2145	13.2%	0.0004
Male		2067/3852	53.7%	1115/2144	52.0%	0.2185
Multiple gestation		1254/3853	32.5%	525/2145	24.5%	<0.0001
Cesarean delivery		2681/3853	69.6%	1413/2145	65.9%	0.0031
Antenatal steroids		3328/3852	86.4%	1807/2145	84.2%	0.0227
Apgar score at 5 min	3 or less	461/3824	12.1%	322/2126	15.1%	<0.0001
	4–7	1464/3824	38.3%	872/2126	41.0%	–
	8–10	1899/3824	49.7%	932/2126	43.8%	–
Outborn		477/3853	12.4%	171/2145	8.0%	<0.0001
Prenatal care		3654/3849	94.9%	2017/2137	94.4%	0.362
Maternal age (years)	20 or less	199/3852	5.2%	185/2145	8.6%	<0.0001
	21–30	1624/3852	42.2%	1046/2145	48.8%	–
	31–40	1841/3852	47.8%	826/2145	38.5%	–
	41+	188/3852	4.9%	88/2145	4.1%	–
Maternal hypertension		826/3846	21.5%	588/2143	27.4%	<0.0001
Chorioamnionitis		404/3846	10.5%	295/2143	13.8%	0.0002
Maternal diabetes		351/3846	9.1%	197/2143	9.2%	0.932

^a^Birth weight was not included in models.

^b^*P* value for chi-square test.

**Table 5:** Estimate of the impact of risk factors on observed difference in Black vs White neonatal mortality^a^.

	Observed mortality	Risk adjusted mortality	Adjustment difference	
White	15.9	16.7	0.8	
Black	16.6	13.7	2.9	
Difference	0.7	3.0	3.7	

Risk factor		Risk impact	Contribution	
Gestational age in weeks		2.41%	65.2%	Increased risk in Black infants
Apgar score at 5 min		0.91%	24.6%	
Small for gestational age		0.29%	8.0%	
Maternal age		0.16%	4.2%	
Location of birth		0.13%	3.5%	
Antenatal steroids		0.12%	3.3%	
Prenatal care		0.00%	0.0%	
Maternal diabetes		0.00%	0.0%	
Chorioamnionitis		−0.01%	−0.2%	Decreased risk in Black infants
Male		−0.03%	−0.9%	
Cesarean delivery		−0.05%	−1.4%	
Maternal hypertension		−0.10%	−2.8%	
Multiple gestation		−0.13%	−3.4%	
Endowment explanation		3.70%	100.0%	
Impact explanation (outborn)		−0.10%		
Estimated difference after risk adjustment		3.60%		

^a^Estimates based on the Blinder–Oaxaca decomposition procedure.

## Discussion

In evaluating the rate of neonatal mortality in very preterm infants, there is no agreed upon standard cohort selection. Researchers have investigated outcomes in cohorts selected on the basis of birth weight, gestational age, time of death, and the presence of severe congenital anomalies. The first goal of our study was to systematically assess the impact of four standard cohort selection approaches on the estimation of very preterm mortality. Our second goal was to assess if cohort section had an impact on the extent of both observed and risk adjusted potential differences in the rates of Black and non-Hispanic White mortality. We observed that compared to our findings of essentially no Black White mortality disparity in observed rates, there was a significantly lower Black as compared to NHW mortality in risk adjusted rates. A third goal for the project emerged and we then employed the Oaxaca decomposition approach in an attempt to understand the potential drivers of this finding.

Our comparison of several cohort selection criteria found that choice greatly affects the estimation of the rate of very preterm neonatal mortality when all races and ethnicities were included (Table 1) and when the cohort was limited to only Black and non-Hispanic White infants (Appendix Table 1). Depending upon the selection criteria, mortality could range from a low of 6.2% (VLBW infants excluding severe congenital anomalies, delivery room and first 12-h deaths) to a high of 16.0% (22–29 weeks including all deaths without serious congenital anomalies), a greater than 2.5-fold increase. If we include infants with congenital anomalies, the rate increases to 17.4%. This stresses the importance of assuring that similar cohort selection criteria have been selected when comparing outcomes across, facilities, networks, regions, and countries. It also stresses the importance of understanding what proportion of potential births and deaths have been included when investigating outcomes in very preterm infants. For example, while the justification for not including infants less than 24 weeks GA, those who have died in the delivery room or first 12 h of life, or those with severe congenital anomalies, is to have a more homogeneous leveled playing field for making comparisons and seems quite reasonable, this selection includes only 56% of the births and 38.5% of the deaths observed when very preterm births are selected as all VLBWs born at greater than 22 weeks. There is also the possibility that if a network of NICUs had important site differences in the immediate postpartum care of periviable infants, these differences would not be detected when deaths in the delivery room and first 12 h of life are not evaluated.

It may be appropriate to not have a single standard approach because estimating the rates of neonatal mortality or the extent of racial/ethnic differences in the rates of neonatal mortality in very preterm infants is dependent upon the information’s specific use. There are at least four different perspectives, each generating data specific to the clinician, the quality improvement officer, the perinatal epidemiologist, and the individual and family. For the clinician, the observed rate reflects the work that will be required, under the assumption that mortality is a proxy for acuity. Although standard cohort selection decisions can profoundly influence the observed rate, the clinician is tasked with caring for the complete birth cohort. So, from the clinical perspective, the total neonatal mortality for the entire very preterm cohort including delivery room, first 12 h, as well as deaths from congenital anomalies may be their most useful estimate (Table 1, All VLBW including congenital anomalies).

For the performance quality officer, it is essential to risk adjust comparisons across institutions to account for differences in case mix, the goal being to assure participants that they have been assessed on “a level playing field.” Previous study has shown that performance rankings may be minimally impacted by varying definitions and risk adjustment strategies.^21^ However, leveling the playing field may require stratification as well as risk adjustment. Because there may be differences in a NICU’s approach at the lower limits of viability, setting a lower limit stratification at 24 weeks of gestation (a point where there is little to no controversy on the appropriateness of performing active resuscitation and initiating comprehensive intensive care) is often seen (Table 1, GA 24–29 weeks).^22^ Furthermore, because of the analytic complexity of assessing risk of death across the mix of serious congenital anomalies, death from congenital anomalies is also often excluded. However, several large databases such as the Vermont Oxford Network and CPQCC include congenital anomalies in their risk adjustment categorized into specific groupings based on each anomalies’ observed mortality.

To the perinatal epidemiologist, a closer look at the risk adjustment model is required to identify those potentially modifiable factors that could be incorporated into interventions, with the caveat that these risk factors may only be associations rather than causalities. Concern over racial /ethnic differences in both preterm morbidity and mortality has increased.^3,23^ We found that although cohort selection had an important impact on estimating the observed rate of neonatal mortality across all selection criteria (study goal one), a difference in observed Black vs White neonatal mortality was seen only in the all deaths VLBW cohort (study goal 2). However, with risk adjustment, an increase in the rate of White and a decrease in the rate of Black neonatal mortality was observed. An apparent Black infant survival advantage was seen across all four selected cohorts (RR 0.83, CL 0.59–0.81) to (RR 0.85, CL 0.72–0.99) that persisted when delivery room deaths and deaths in the first 12 h were excluded (RR 0.70, CL 0.61–0.80) to (RR 0.73, CL 0.63–0.85). The finding of a statistically significant Black survival advantage in very preterm mortality that persisted across cohort selection criteria was not unique to our study. In a study of the quality of care provided in 737 member hospitals of the Vermont Oxford network, Edwards and associates reported that excluding infants less than 25 weeks, congenital anomalies, delivery room and deaths within 12 h, the risk adjusted survival component of the Baby-MONITOR was significantly higher for Black infants when based on within hospitals analysis. They also report that this advantage was also present when the analysis was conducted in each of the country’s 9 U.S. census divisions. With respect to the possible impact of cohort selection, similar to what we observed, the survival advantage for Black infants persisted when delivery room and deaths within the first 12 h of life were included in the analytic cohort.^24^ Additionally, in a study based on 20,092 infants to at GA 22 0/7 to 27 6/7 weeks with a birth weight 401–1500 g cared for at member hospitals of the NICHD neonatal research network which excluded infants who died in the first 12 h of life and those with severe congenital anomalies, Travers et al. reported “that in contrast to the unadjusted mortality rates, adjusted mortality rates favored Black infants.”^4^ However, when estimating the extent of Black–non-Hispanic White neonatal disparity it is also important to consider timing of death. The survival advantage that we found for Black infants at 22–29 weeks has been reported to be lost around 32–33 weeks and then reversed as infants are born closer to term.^8^

To explore the contradiction that we and others have observed of no to minimal racial difference in the observed neonatal mortality and an apparent Black infant survival advantage after risk adjustment, we performed a Blinder-Oaxaca decomposition.^4,24^ The decomposition partitions out the extent of the difference in risk adjusted rates of two groups that is due to differences in the percentage of each risk factor (endowment) and due to differences in the strength of association of each endowment with the outcome. Our models included biologic variables, potentially modifiable variables, maternal health conditions, maternal care factors, and the infant’s condition at birth. Although our models only describe strength of association, they do offer potential areas of intervention. Low gestational age in Black infants accounted for 65.2% of the difference in adjusted rates. This finding supports the importance of developing effective strategies to reduce preterm delivery. The second largest contributor was condition at birth as reflected by low 5-min Apgar score (24.6%). Condition at birth is influenced by maternal health status as well and the quality of prenatal and delivery care. Initiatives to identify and effectively treat diabetes and hypertension as well as to assure equity in the quality of clinical care, for example, by assuring that all eligible are treated with antenatal steroids (3.3% of the difference) have the potential of improving condition of the infant at birth. Although a detailed discussion of the potential interventions suggested by our analysis is beyond the scope of this discussion, the decomposition shown in Table 5 provides estimates of the strength of association of each risk factor. After assessing each factor’s potential malleability, this information could be useful in prioritizing the need for the development of new interventions and the prioritization of existing interventions.

The apparent advantage in neonatal mortality seen by Black infants, when observed in other settings, has been debated as to whether it represents a true biological advantage,^25^ is a result of stratification bias,^26^ or arises from important but as yet unidentified risk factors unique to or more prevalent in White infants.^27^ While these issues are important to the clinician and epidemiologist, they fail to fully address the concern of a pregnant individual, “What is the likelihood that my fetus will survive and be healthy?” From an equity perspective, a Black family may ask “If I were to have a baby, what is the likelihood that the baby will survive compared to that of a baby born to a White family?” To get insight into this concern, we utilized the postnatal modification of the fetus at risk approach as suggested by Basso.^28^ Simply stated, it is the percentage of infants that are expected to be born very preterm multiplied by their rate of survival. In our California birth cohort, 1.51% of 131,667 Black infants vs 0.45% of 683,199 White non-Hispanic infants were born between 22 and 29 weeks of gestation. Our crude estimate would be the RR of extremely preterm birth (1.51/0.45 = 3.36) times the RR of death as a Black extremely preterm infant born at 22–29 weeks (0.84, Table 3), giving an overall disparity of 2.8 (3.36 × 0.84). So, for a Black individual, the approximate likelihood that their baby will be born extremely prematurely and die before leaving the hospital is 2.8 times greater than a baby born to a White non-Hispanic individual. Although the fetus at risk approach and its modification is not universally accepted,^28^ it focuses attention on the notion that burden to the clinician, as well as the family, is dependent upon both rate and volume.^29^ Similar findings were reported by Wu et al. who state that this paradox (the Black disadvantage estimated by the fetus at risk approach) seen when GA specific neonatal mortality rate is stratified by risk factors such as race, “may be explained if more neonates without congenital anomalies are born at earlier GA among Black vs White mothers.”^30^ Our study of very preterm infants is limited to California, which may have population and healthcare-specific differences from other states. For example, our 2020 infant mortality of 3.92 is one of the lowest in the country.^8^ Although a similar risk adjusted very preterm mortality advantage was found for Black infants in NICUs aggregated across the nine US census regions,^24^ the survival advantage seen in California may not hold true across all United States NICUs. Regional differences exist in regard to distribution of patients by race and ethnicity across NICUs^31^ as well as statewide differences in neonatal mortality and Black-White disparities by birth weight,^32^ which may impact mortality estimates. From an equity perspective, it is also important to point out that summary findings across large regions and networks do not reflect the extent of disparity that could be encountered at some of their member facilities.^4^ Additionally, our overall California findings may not hold up in regions such as New York City where Black births are concentrated in lower performing hospitals.^33^

In conclusion, although cohort selection can result in an almost three-fold difference in the rate of very preterm neonatal mortality, when cohort selection is designed for a specific purpose, it can provide useful estimates. In California, statistically significant Black–non-Hispanic White disparity was only seen in the risk adjusted rates of very preterm mortality, there being a Black survival advantage that was similar across all of our selected cohorts. Decomposing the difference in rates between these two populations had the potential of revealing driving differences in their risk profiles including the higher rate of extremely preterm births, the lower delivery of antenatal steroids, and poorer condition at birth in Black infants that could suggest opportunities for improvement.

## Data Availability

The data that support the findings of this study are available from the California Perinatal Quality Care Collaborative but restrictions apply to the availability of these data, which were used under license for the current study, and so are not publicly available. Data are, however, available from the California Perinatal Quality Care Collaborative through an application process.

## Appendix

**Table 6:** Combined Black and non-Hispanic White neonatal mortality by cohort selection criteria.

	# infants	Delivery room death		Died 12 h^a^		Died 13 h– discharge^b^		All deaths	
		*n*	%	*n*	%	*n*	%	*n*	%
24–29 weeks gestational age	5353	55	1.0%	57	1.1%	391	7.3%	503	9.4%
Add 22–<24 weeks	645	299	46.4%	26	4.0%	140	21.7%	465	72.1%
22–29 weeks gestational age	5998	354	5.9%	83	1.4%	531	8.9%	968	16.1%
Add 401–1500 g	3063	71	2.3%	4	0.1%	27	0.9%	102	3.3%
401–1500 g or 22–29 weeks (“VLBW cohort”)	9061	425	4.7%	87	1.0%	558	6.2%	1070	11.8%
Add congenital anomaly 401–1500 g or 22–29 weeks	639	425	4.7%	87	1.	300	17.5%	621	36.2%
401–1500 g or 22–29 weeks, “VLBW cohort” including congenital anomaly	9700	509	5.2%	110	1.1%	675	7.0%	1294	13.3%

^a^Died in the first 12 h after surviving the delivery room.

^b^Died prior to discharge after surviving the first 12 h after birth.

**Table 7:** Decrease in cohort size used for the Blinder–Oaxaca decomposition (OX) due to 1 or more missing risk variables.

	All	OX	dif	%
Black	2145	2114	31	1.4%
NHW	3853	3812	41	1.1%
Total	5998	5926	72	1.2%

**Table 8:** Oaxaca–Blinder analysis of Black and non-Hispanic White difference in adjusted neonatal mortality.

	*N* used	Mortality rate			
Black	2114	0.161779			
Non-Hispanic White	3812	0.156873			
Difference	–	0.004906			
Explained	–	0.035979			
Unexplained	–	−0.031074			

Contributions from racial differences in:					
Explained		Coeff	SE	*P*	% Explained
Gestational weeks		0.0234423	0.003487	<0.0001	65.2%
Small for gestational age		0.0028671	0.000967	0.003	8.0%
Multiple gestation		−0.0012376	0.000987	0.21	−3.4%
Apgar score at 5 min		0.0088611	0.001951	<0.0001	24.6%
Male		−0.0003285	0.000329	0.319	−0.9%
Location of birth		0.0012764	0.001121	0.255	3.5%
Prenatal care		6.25E−06	0.000845	0.941	0.0%
Cesarean delivery		−0.0005093	0.00037	0.169	−1.4%
Maternal age		0.0015089	0.000989	0.127	4.2%
Antenatal steroids		0.0011694	0.00079	0.139	3.3%
Chorioamnionitis		−6.43E−05	0.000424	0.879	−0.2%
Maternal diabetes		−8.67E−06	0.000115	0.94	0.0%
Maternal hypertension		−0.0010038	0.000799	0.209	−2.8%
Unexplained:					
Gestational age weeks		0.3475707	0.330569	0.293	
Gestational age weeks^2^		–	–	–	
Small for gestational age		0.005707	0.005138	0.267	
Multiple gestation		0.0015094	0.007674	0.844	
Apgar score at 5 min		0.0289592	0.038569	0.453	
Male		−0.0069873	0.011265	0.535	
Location of birth		−0.0091849	0.005101	0.072	
Prenatal care		0.0124302	0.049043	0.8	
Cesarean delivery		−0.0025717	0.016771	0.878	
Maternal age		−0.0800082	0.061009	0.19	
Antenatal steroids		−0.0365817	0.027675	0.186	
Chorioamnionitis		−0.0033971	0.003636	0.35	
Maternal diabetes		−0.0068467	0.00505	0.175	
Maternal hypertension		−0.0022598	0.009991	0.821	
_cons		−0.279413	0.314432	0.374	
